# Bio-conjugated nanoarchitectonics with dual-labeled nanoparticles for a colorimetric and fluorescent dual-mode serological lateral flow immunoassay sensor in detection of SARS-CoV-2 in clinical samples†

**DOI:** 10.1039/d3ra04373h

**Published:** 2023-09-11

**Authors:** Sang Ki Kim, Jong Uk Lee, Myeong Jin Jeon, Soo-Kyung Kim, Sang-Hyun Hwang, Min Eui Hong, Sang Jun Sim

**Affiliations:** a Department of Chemical and Biological Engineering, Korea University 145, Anam-ro, Seongbuk-gu Seoul 02841 Republic of Korea simsj@korea.ac.kr; b Department of Chemical Engineering, Sunchon National University 225 Jungang-ro Suncheon Jeollanam-do 57922 Republic of Korea; c Department of Laboratory Medicine, Ewha Womans University Mokdong Hospital Seoul 07985 Republic of Korea; d Department of Laboratory Medicine, Asan Medical Center, University of Ulsan College of Medicine Seoul 05505 Republic of Korea; e Business Development, Kyung Nam Pharm.Co.,Ltd 702 Eonju-ro Gangnam-gu Seoul 06061 Republic of Korea

## Abstract

Serological detection of antibodies for diagnosing infectious diseases has advantages in facile diagnostic procedures, thereby contributing to controlling the spread of the pathogen, such as in the recent SARS-CoV-2 pandemic. Lateral flow immunoassay (LFIA) is a representative serological antibody detection method suitable for on-site applications but suffers from low clinical accuracy. To achieve a simple and rapid serological screening as well as the sensitive quantification of antibodies against SARS-CoV-2, a colorimetric and fluorescent dual-mode serological LFIA sensor incorporating metal-enhanced fluorescence (MEF) was developed. For the strong fluorescence signal amplification, fluorophore Cy3 was immobilized onto gold nanoparticles (AuNPs) with size-controllable spacer polyethyleneglycol (PEG) to maintain an optimal distance to induce MEF. The sensor detects the target IgG with a concentration as low as 1 ng mL^−1^ within 8 minutes. The employment of the MEF into the dual-mode serological LFIA sensor shows a 1000-fold sensitivity improvement compared with that of colorimetric LFIAs. The proposed serological LFIA sensor was tested with 73 clinical samples, showing sensitivity, specificity, and accuracy of 95%, 100%, and 97%, respectively. In conclusion, the dual-mode serological LFIA has great potential for application in diagnosis and an epidemiological survey of vaccine efficacy and immunity status of individuals.

## Abstract 2

A colorimetric-fluorescent dual-mode lateral flow immunoassay sensor incorporating metal-enhanced fluorescence (MEF) for the rapid and sensitive serological detection of SARS-CoV-2 IgG was developed.

## Graphical abstract

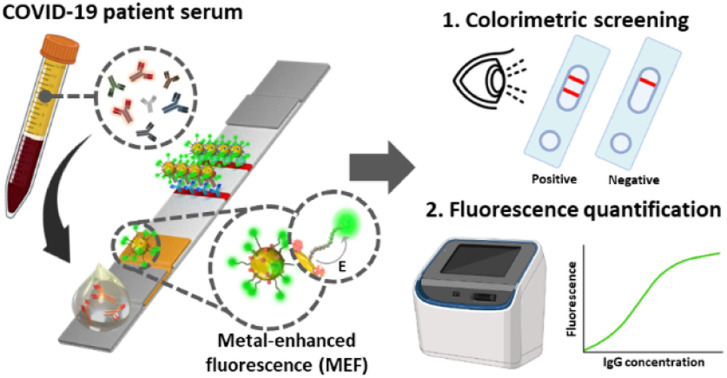

## Introduction

First identified in late 2019, the pandemic of COVID-19, which is caused by a novel virus designated as SARS-CoV-2, is an urgent public health threat owing to its exceptionally rapid infection and numerous mortalities.^1^ As the pandemic continues, the current standard method for diagnosing COVID-19 is the reverse transcription polymerase chain reaction (RT-PCR).^2^ However, the RT-PCR method is generally limited in its wide application due to the need for expensive equipment, highly skilled personnel and a time-consuming procedure. For these reasons, serological assays to detect immunoglobulin M (IgM)/immunoglobulin G (IgG) antibodies against SARS-CoV-2 have been proposed as an alternative method for diagnosing COVID-19.^3^ Serological testing is simple and rapid because it does not require isolation or amplification of the biomarkers from the specimen.^4^ These features are especially valuable for diagnosing highly infectious pathogens, which require prompt control and management to prevent the spread of the disease.^5–7^

Among the many serological techniques for monitoring and diagnosing infectious diseases, such as COVID-19, lateral flow immunoassay (LFIA) sensors have been used due to the user-friendly procedures and straightforward test results.^8^ Despite these advantageous features, the conventional LFIA sensor based on colorimetric readouts has limited sensitivity due to the insufficient brightness of the reporter, namely gold nanoparticles (AuNPs) and is prone to observer bias for ambiguous test results.^9–11^ On the other hand, the recently developed fluorescence-based LFIA sensor has shown higher sensitivity, lower background noise and easier quantitative detection than its colorimetric counterpart.^12–15^ However, the lack of visual signal from the LFIA sensor poses challenges for large-scale screening.^16,17^ For these reasons, integrating colorimetric and fluorometric assays into one test strip is a promising approach to simultaneously achieving rapid detection and sensitive quantitation.^18^

**Fig. 1:**
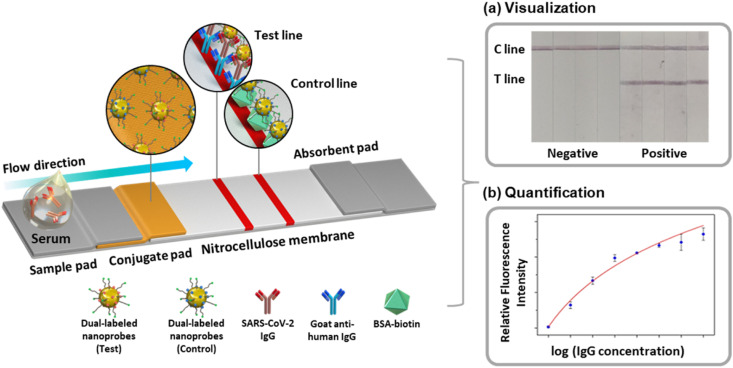
Schematic of the MEF-based dual-mode serological LFIA sensor for SARS-CoV-2 IgG detection. Examples of (a) color-based visual detection and (b) fluorescence-based quantification.

In this study, we present a colorimetric and fluorescent dual-mode serological LFIA sensor for rapid, accurate, sensitive, and specific detection of antibodies against SARS-CoV-2 from a patient's serum. In particular, we introduced the gold nanoprobes coupled with fluorophores to achieve both naked-eye screening and improved fluorometric sensitivity (Fig. 1). The gold nanoprobe provides a clear colorimetric signal and amplifies the fluorescence of proximal fluorophores by transfer of electromagnetic energy, which is called the metal-enhanced fluorescence (MEF) phenomenon.^19,20^ Adapting the MEF in the LFIA sensor can lead to several orders of magnitude lower detection limits of the conventional fluorescence LFIA-based diagnosis.^20^ The dual-mode serological LFIA sensor can identify the SARS-CoV-2 IgG antibody as a model analyte in serum within 8 minutes with a 1 ng mL^−1^ detection limit, which is 1000-fold more sensitive than the colorimetric LFIA sensor. Serological detection of IgG in clinical samples, including 43 COVID-19 patients and 30 healthy controls, demonstrated that the MEF-based dual-mode serological LFIA achieved a diagnostic accuracy of over 97%. Thus, this dual-mode approach for serological LFIA provides a promising strategy for on-site, fast, and accurate diagnosis of various contagious diseases not limited to the SARS-CoV-2 virus, which helps prevent and control disease spread.

## Results and discussion

### Characterization and optimization of dual-labeled nanoprobes

Fig. 1 is the schematic of the dual-mode serological LFIA developed in this paper. The nanoprobes were dual-labeled with a fluorophore and target-specific antigens and loaded on the conjugate pad. As the serum containing the target antibody is dropped onto the kit, the nanoprobes react with the antibody to form nanoprobe-antibody conjugate. The conjugate flows through the kit *via* capillary force exerted by the nitrocellulose membrane (NCM) until captured from the secondary antibody immobilized on the test line.^9,10^ The dual-labeled nanoprobes simultaneously produce both colorimetric and fluorescence signals, which are exploited for naked-eye screening and target quantification.

**Fig. 2:**
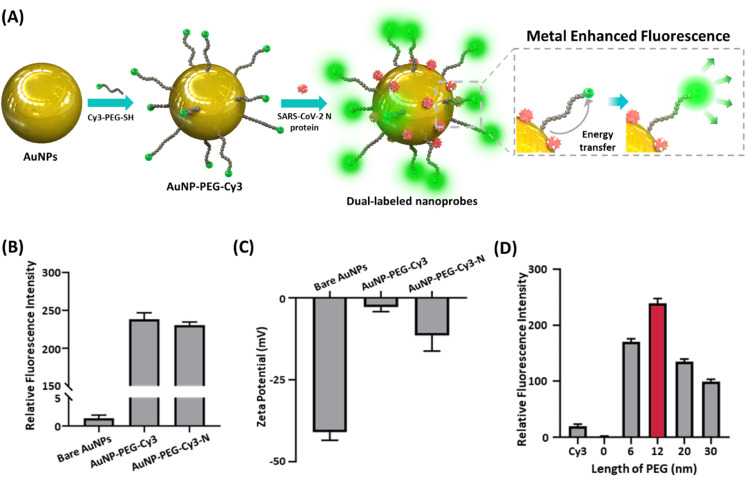
Characterization of dual-labeled nanoprobes. (A) Construction of nanoprobes and mechanism of MEF. (B) Fluorescence intensity and (C) zeta potential of bare AuNPs, AuNP-PEG-Cy3 and dual-labeled nanoprobes. (D) Fluorescence intensity with various PEG lengths for effective MEF.

To achieve the visual detection and fluorescent quantification in the same kit, the AuNP having drastic plasmonic properties was adopted for the nanoprobe. According to the Mie theory regarding the scattering of the nanoparticles, the AuNPs with a size near 40 nm induce exceptionally strong light scattering and absorption, generating a remarkable color signal in the visible wavelength.^21,22^ In addition, when the localized surface plasmon resonance (LSPR) spectrum of the AuNP overlaps with the spectrum of the fluorophore, the energy transfer from the excited AuNP to the fluorophore (Fig. 2A).^19,23^ Herein, the Cy3 was selected as the fluorophore for the dual-labeled nanoprobe, which has great spectral overlap with the AuNP.^24^ The AuNPs were synthesized *via* citrate reduction^25^ and characterized by transmission electron microscope (TEM), UV-vis spectrometry, nanoparticle tracking analysis (NTA), Fourier transform infrared (FT-IR) and X-ray diffraction (XRD) (Fig. S1 and S2†). The TEM image and UV-vis spectrum showed that the synthesized AuNPs have approximately 40 nm diameters (Fig. S1A and S1B†). NTA measurement is consistent with these measurements, with a highly homogeneous size distribution (Fig. S1C†).

To evaluate the MEF effect, the interval between the AuNP and Cy3 was tailored by using the polyethyleneglycol (PEG) molecule, which has high rigidity, as a molecular spacer between AuNP and Cy3 with adjustable length (Fig. 2A).^26^ The immobilization of Cy3-PEG and antigen protein on the nanoparticle was confirmed with the change of fluorescence intensity and zeta-potential (Fig. 2B and C). The new peaks at 1060, 1100 and 1655 cm^−1^ from FT-IR spectra (Fig. S2A†) and the strong reflection of the XRD pattern due to the PEG (Fig. S2B†) also confirm the successful modification of AuNP with PEG-Cy3 and antigen protein. Microplate reader allowed measuring the fluorescence intensity of nanoprobes within the emission wavelength range, including peak position (568 nm) in varying nanoprobe concentration (Fig. S10†).^27^ The measured fluorescence intensities of the nanoprobes with varying lengths of PEG molecules (Fig. 2D) revealed that the AuNPs conjugated with 12 nm Cy3-PEGs expressed the most drastic fluorescence signal, with 10 times higher intensity than the unconjugated Cy3, proving remarkable enhancement from MEF. As the distance between the AuNP and Cy3 became shorter than 12 nm, the Förster resonance energy transfer (FRET) from the Cy3 to the AuNP caused the fluorescence quenching,^26,28^ suppressing the signal down to virtually zero when Cy3 was directly attached to the AuNP. These outcomes suggest that the distance between the Cy3 and the AuNP can be adjusted and maintained by utilizing PEG as a molecular spacer to produce an exceptional MEF effect in our nanoprobe conformation. Furthermore, the selection of the target antibody was based on the binding affinity to the antigen, which led to a more accurate diagnosis.^3,29^ Fig. S3 and S4† regarding the binding affinities of the antibodies to the nanoprobes conjugated with major SARS-CoV-2 antigens – N, spike (S), spike subunit 1 (S1), subunit 2 (S2) and receptor binding domain (S-RBD) – affirm that the N protein antibody showed superior interaction against the antigen. Combined with the high immunogenicity and the low mutation rate of the N protein,^3^ this high binding affinity makes the IgG against the N protein an ideal target for diagnosing SARS-CoV-2 infection.

### Evaluation of the analytical performance of the MEF-based dual-mode serological LFIA sensor

**Fig. 3:**
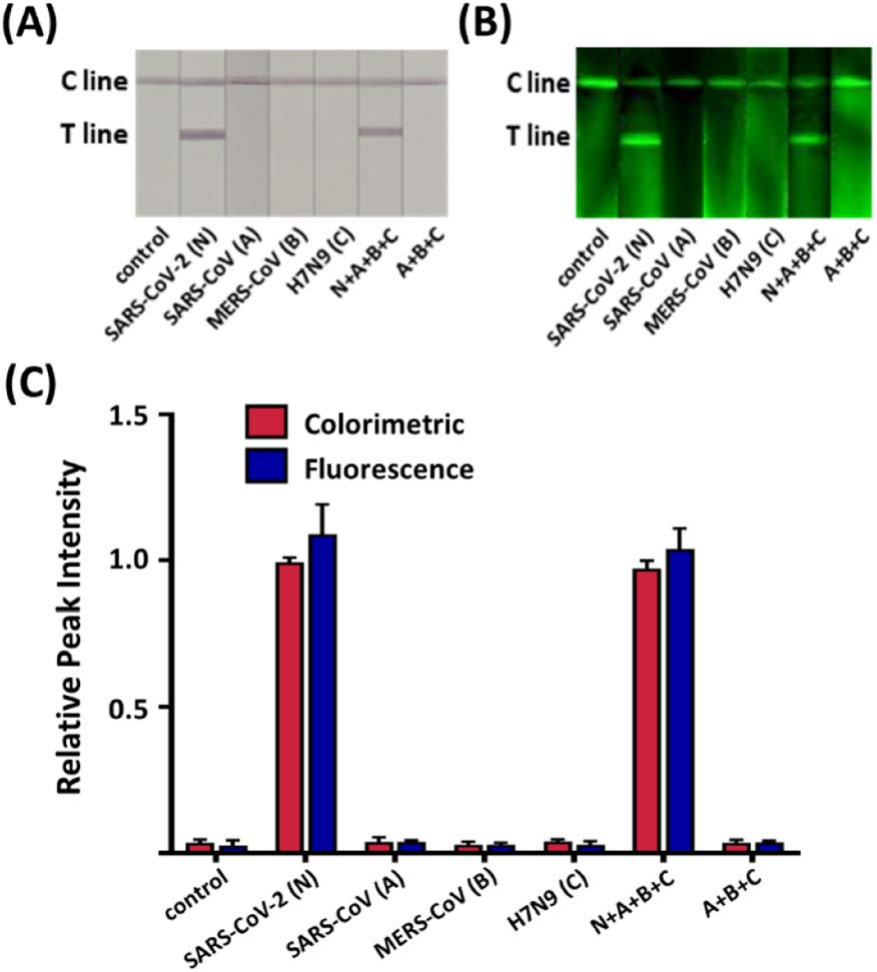
Specificity tests of the MEF-based dual-mode serological LFIA with various interfering analytes, including SARS-CoV, MERS-CoV, and H7N9 antibodies. The concentration of the target antibody is 1 μg mL^−1^, whereas the other proteins are 100 μg mL^−1^. (A) Colorimetric images, where C and T lines denote the control and test line, respectively. (B) Fluorescent images of the same strips. (C) Relative peak intensity (RPI) of color and fluorescence signals. Error bars indicate the standard deviation from three independent experiments.

The assessment of the specificity of our serological LFIA sensor was conducted on the serum sample, including the IgG against several major endemic viruses, namely severe acute respiratory syndrome coronavirus (SARS-CoV), middle east respiratory syndrome coronavirus (MERS-CoV), and avian influenza (H7N9) along with the SARS-CoV-2. As indicated in Fig. 3, all non-specific samples exhibited negligible signals in both colorimetric and fluorescent assays comparable to the blank negative. Conversely, significantly higher colorimetric and fluorometric signals were observed when the SARS-CoV-2 IgG was loaded, despite a 100-fold lower concentration than non-specific antibodies. The sensor also displayed identical signal intensity even when the non-target IgG was present with the target. To sum up, our sensor precisely recognizes the target IgG *via* the antigen–antibody interaction without interfering with other non-target antibodies in the serum.

**Fig. 4:**
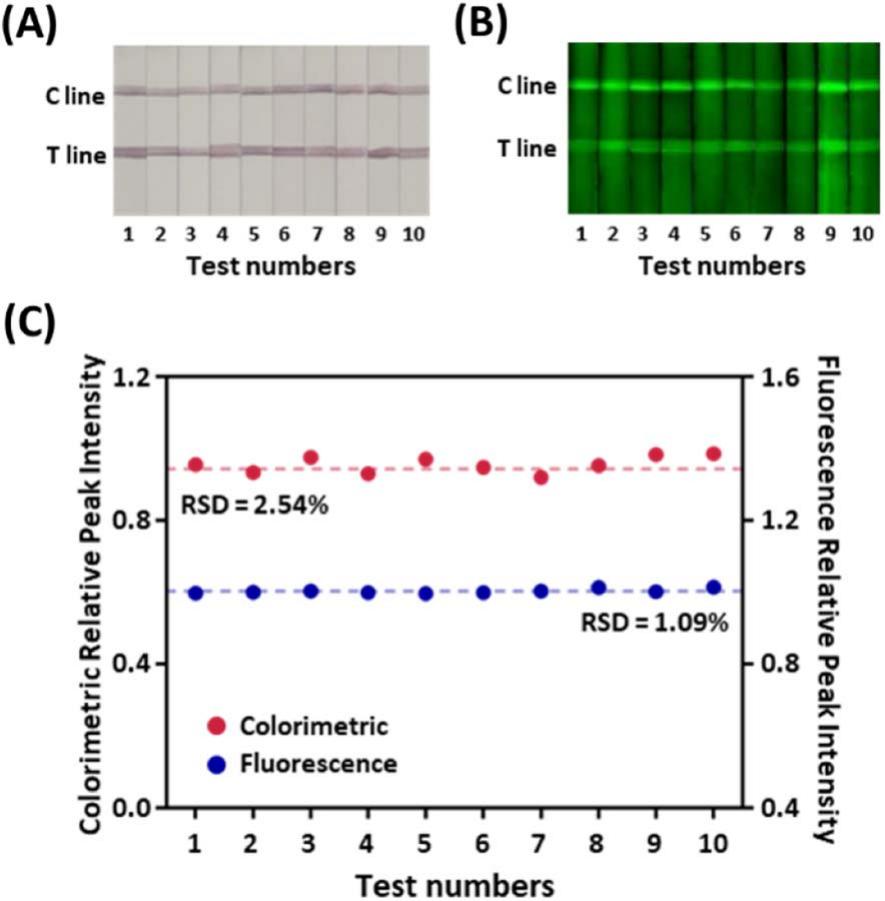
Reproducibility tests of the MEF-based dual-mode serological LFIA by loading the same samples ten times. (A) Colorimetric and (B) fluorescent images of the LFIA strips. (C) RPI of color and fluorescence signals.

Traditionally, one of the most severe drawbacks of LFIA-based serological diagnosis has been the lack of reproducibility.^30^ The low consistency in the inter-batch test results mainly arose from the irregularity of manufacturing probes and strips, impeding the application of the LFIA sensor in the clinical field. Especially the fluorescence-colorimetric LFIA sensors tend to employ bead-based nanoprobes, including colorimetric particles and fluorophores.^31,32^ However, the bead-based nanoprobes suffer from low signal reproducibility because the immobilization of the signal-inducing species occurs irregularly on the beads.^33^ The strip-to-strip reproducibility of the signal in our LFIA sensor was verified for the strips from 10 random batches. Fig. 4A and B display distinctive red lines with green fluorescent signals in all strips. When 1 μg mL^−1^ of the target was applied to the sample pad, the relative standard deviation (RSD) of the relative peak intensities (RPIs), the signal intensity ratio of the T and C line from the tested strips was 2.54% in color contrast and 1.09% in fluorescence (Fig. 4C). Even in the lower concentration of target IgG (100 ng mL^−1^ and 10 ng mL^−1^), the RSDs of the test results maintained below 5% (Fig. S5†), although the lower target concentration often leads to more imprecise measurements due to the sampling noise and imperfect target recognition.^34^ The consistent signal intensities imply that the nanoprobes are produced in homogeneous quality to have consistent signal outputs owing to the rigid PEG spacers, guaranteeing the reproducibility and reliability of the test results.

For the practical application of serological LFIA sensors, consistent test results from identical samples are essential during the whole shelf life. Notably, the fluorophore is vulnerable to photobleaching which can hamper the fidelity of the diagnosis in the extended shelf life.^35,36^ We examined the reliability of the signal over a prolonged storage time of up to 180 days. The LFIA sensors were fabricated and stored at room temperature until testing the performance of the sensors. Fig. S6† confirms that no noticeable changes were observed in the intensity of the colorimetric results. Fluorescence intensity also remained high after six months without remarkable fluorescence bleaching during the tested period. The enhanced photostability is mainly because the faster fluorescence emission caused by the MEF prevents the light-induced disintegration of the fluorophore.^37^ This result confirmed that our dual-mode serological LFIA sensor incorporating the MEF-based nanoprobe has an elongated shelf life of over 6 months in the ambient temperature.

**Fig. 5:**
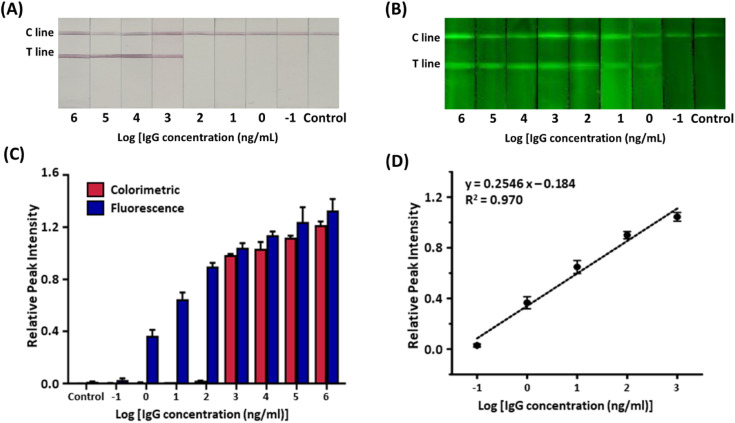
Sensitivity of the MEF-based dual-mode serological LFIA. Serially diluted target antibodies (1000 μg mL^−1^ to 100 pg mL^−1^) were tested. (A) Colorimetric and (B) fluorescent images. (C) Bar graph showing the relative peak intensity of color contrast and fluorescence signal. (D) Calibration curve for SARS-CoV-2 IgG spiked in serum by the relative peak intensity of fluorescence signals.

Recent studies have suggested that every 10-fold decrease in the limit of detection (LOD) reduces the false-negative rate of a test by 13%.^38^ Since false-negative diagnostic results gravely affect the spread of infectious diseases, diagnostic assays need to have low LODs.^39^ The colorimetric and fluorometric sensitivity of the serological LFIA sensors were evaluated with serially diluted target IgG in serum (Fig. 5A and B). In the colorimetric assay under ambient light, the red band started to develop when the target concentration was 1 μg mL^−1^ (Fig. 5C). Although the colorimetric sensitivity can be improved by various strategies,^40,41^ the colorimetric sensitivity of our serological LFIA is sufficient for clinical settings, considering the typical level of IgGs in blood samples for COVID-19 (>10 μg mL^−1^).^42^ Meanwhile, in the fluorometric assay, the green emission of the test lines was observed with a target concentration as low as 1 ng mL^−1^ (Fig. 5D), which is a 1000-fold lower detection limit than the colorimetric measurement. The LOD of our serological LFIA sensor is outstandingly lower than previously reported studies (Table S1†). Compared with these literature, the dramatic MEF phenomenon induced by the AuNP-Cy3 structure linked with rigid PEG spacer greatly contributes the improved fluorescence intensity and thus the sensitivity. The combination of the colorimetric and fluorometric assays enabled rapid color-based screening and highly sensitive quantification in one testing strip, effectively overcoming the low accuracy of the LFIA sensor. Also, while the colorimetric signal works “on-off”, the fluorometric signal intensities show concentration-dependent behavior with the linear dynamic range of 1–1000 ng mL^−1^ (Fig. 5E). In this concentration range, the linear regression equation is: *y* = 0.2546 log *x* − 0.184, *R*^2^ = 0.970 where *x* is IgG concentration. The linear regression model demonstrated an excellent correlation (*R*^2^ > 0.95) between the RPI and logarithm of the target IgG concentration. From the advanced LOD and linear dynamic range of the sensor, we anticipate that our sensor can diagnose asymptomatic patients who are reported to have lower IgG expression in sera than in symptomatic groups.^43^

### Clinical evaluations of the MEF-based dual-mode serological LFIA sensor

The feasibility of our MEF-based dual-mode serological LFIA sensor for detecting SARS-CoV-2 antibodies was verified by testing 73 serum samples, including 43 positive and 30 negative serum specimens (Table S2†). The positive samples could be divided into two sub-groups with the date of sample acquisition, one (Patient No. P1-P15) in mid-2021 and another (P16-P43) in early 2022. As illustrated in Fig. S7,† the colorimetric and fluorescent signals were detected in 39 and 41 of the COVID-19 patient's serum samples (Fig. S7A and S7B†). Also, in the case of negative serum samples, no significant colorimetric and fluorometric signals were observed (Fig. S7C and S7D†). Notably, two patient samples that showed false-negative results in colorimetric readout were correctly detected with fluorescence mode in our dual-mode serological LFIA strips, indicating that MEF can effectively compensate for the low true positive rate of the colorimetric LFIA sensors.

**Fig. 6:**
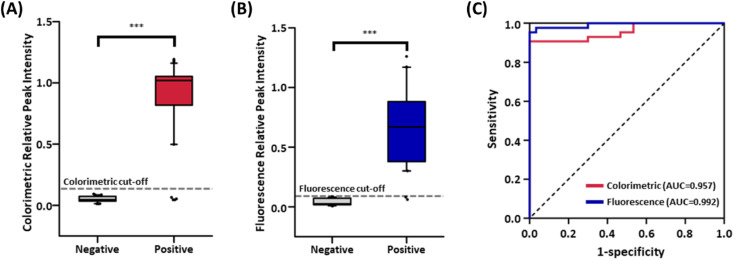
MEF-based dual-mode serological LFIA with 73 clinical samples. (A) Box plot of colorimetric results and (B) fluorescence results for 43 positive and 30 negative samples. (***) indicates *p* < 0.0001 (Student's *t*-test). (C) Receiver operating characteristics (ROC) curve analyses of 73 serum samples to assess the detection capacity of the developed LFIA.

The box plots for average RPI values in colorimetric and fluorescence signals suggest the existence of a clear cut-off between patients and normal controls (Fig. 6A and B; *p* < 0.0001 for both modes). A receiver operating characteristic (ROC) curve was plotted to assess the discrimination ability based on the RPI values (Fig. 6C). The area under the ROC curve (AUC), an index for the discrimination ability of the diagnostic test, was 0.957 for color contrast and 0.992 for fluorescence, exceeding 0.900, which is generally accepted as a highly accurate analysis method.^44^ The ROC curve and Youden's index (*J*) were used to define the cut-off value of the LFIA sensor by assessing clinical sensitivity and specificity at varying thresholds. The optimal cut-off value was calculated using the formula: *J* = max (sensitivity + specificity − 1).^45^ The cut-off values were determined as RPI = 0.093 for color contrast and 0.033 for fluorescence. From these cut-off values, COVID-19 patients and healthy controls were effectively distinguished, having a clinical sensitivity of 90.7% and 95.3% for colorimetric and fluorometric measurement and 100% specificity for both modes. On the contrary, the commercially available ELISA kit showed an AUC of 0.842 (84% sensitivity, 80% specificity) (Fig. S8†), which is lower than our serological LFIA sensor. As a result, the diagnosis of COVID-19 based on SARS-CoV-2 IgG using our serological LFIA sensor accomplished an accuracy of 94.5% and 97.3% for color contrast and fluorescence (Fig. S9†). These results are superior to other reported studies for LFIA-based serological detection of SARS-CoV-2 antibodies, enhancing clinical accuracy with less reaction time (Table S1†). This advanced performance of our sensor originated from the incorporation of plasmonic nanoprobes, which features a distinct colorimetric signal and the dramatically amplified fluorescence signal due to the MEF. Consequently, the proposed serological LFIA sensor holds great potential for accurate serological detection of antibodies for emerging infectious diseases, including the SARS-CoV-2 and inspires future research in the immune responses or vaccination to cope with those disorders.

## Conclusions

In this study, we developed a colorimetric-fluorescent dual-mode serological LFIA sensor for rapid and ultrasensitive detection of antibodies against SARS-CoV-2 in serum. The highly improved sensitivity against the SARS-CoV-2 IgG antibodies was achieved by employing a plasmonic AuNP nanoprobe labeled with fluorophore Cy3 and N protein antigen, providing distinctive colorimetric readouts and a MEF-based intensified fluorescence signal within 8 minutes. This proposed sensor enables more accurate quantitative detection with a 1000-fold higher sensitivity (down to 1 ng mL^−1^) than the colorimetric screening, thereby avoiding the false negative result of traditional colorimetric-based LFIA sensor and ensuring the accuracy and reliability of serological diagnosis. Furthermore, the size-controllable PEG spacer between AuNP and Cy3 gives strong fluorescence signal fidelity in multiple batches of strips, which is desirable for diagnostic applications. The clinical performance of our serological LFIA sensor was validated through an analysis of 73 human serum samples, resulting in sensitivity (90.7% for color, 95.3% for fluorescence), selectivity (100%), and accuracy (97.3%). Thus, the MEF-based dual-mode serological LFIA sensor developed in this study has great potential for developing point-of-care diagnostics enabling accurate and quantitative detection of bioanalytes and is expected to have the capability in vaccine evaluation and epidemiological surveys for COVID-19 and other diverse contagious diseases.

## Experimental methods

### Reagents

Gold(iii) chloride trihydrate (HAuCl_4_·3H_2_O), bis(*p*-sulfonatophenyl)phenylphosphine dihydrate dipotassium salt (BSPP), sodium chloride (NaCl), bovine serum albumin (BSA), sucrose, tween-20, sodium tetraborate, boric acid, BSA-biotin, normal human serum, and goat anti-human IgG (Fc) secondary antibody were purchased from Sigma-Aldrich (St. Louis, MO, USA). Phosphate buffer saline (PBS, pH 7.4, 0.1 M) was provided by Lonza (Basel, Switzerland). Trisodium citrate dihydrate was obtained from Junsei (Tokyo, Japan). Cy3-PEG-thiol (Mw 2000) was purchased from Biochempeg Scientific Inc (Watertown, MA, USA). Five different recombinant SARS-CoV-2 antigen–antibody pairs, namely nucleocapsid (N), spike (S), spike S1 and S2 subunits, and spike receptor-binding domain (S-RBD) were acquired by MyBioSource (San Diego, CA, USA). The component of the LFIA sensor and streptavidin were purchased from Millipore (Billerica, MA, USA). Lateral flow plastic cassette was supplied by DCN Diagnostics (Carlsbad, CA, USA). SARS-CoV-2 (COVID-19) IgG ELISA kits were purchased from Abcam Inc (Cambridge, MA, USA).

### Instrumentation

To evaluate the physical properties of the dual-labeled nanoprobes, TEM, NTA system, zeta potential analyzer, FT-IR, XRD and UV-vis spectrophotometry were used. Microplate reader allowed measuring the fluorescence intensity of nanoprobes within the emission wavelength range, including peak position (568 nm) in varying nanoprobe concentration (Fig. S10†). Fluorescence signals of the test strips were obtained using a Typhoon FLA 9500 scanner at a spatial resolution of 25 μm, with 532 nm laser and 570 BP 20 filter. Photographs of the LFIA strips were captured using a digital camera. The ImageJ software converted the optical densities of the lines to relative peak intensities (RPI), which is the signal intensity ratio of the test and control lines.

### Preparation of dual-labeled nanoprobes

40 nm AuNPs were synthesized using a citrate reduction process.^25^ Briefly, HAuCl_4_·3H_2_O solution (60 mL, 1 mM) was vigorously stirred (1500 rpm) and heated. Trisodium citrate solution (6 mL, 2.27 μM) was added when the solution started to boil at 100 °C. The solution underwent color change into deep red color and was cooled under stirring at 1500 rpm to room temperature (25 °C). After filtration by using 0.22 μm membrane filter, the characteristics of AuNP were confirmed using TEM, UV-vis and NTA (Fig. S1†).

To prepare the dual-labeled nanoprobe, BSPP (0.020 g) was first added to the AuNP solution (10 mL, 0.1 nM) and incubated overnight, followed by centrifugation thrice at 6000 rpm for 40 min.^46^ Cy3-PEG-thiol (15 μL, 200 μg mL^−1^ in DI water) were mixed with BSPP-coated AuNPs (0.22 nM) dispersed in borate buffer (10 mM, pH 9, 1 mL) and incubated overnight with vigorous shaking. After the removal of free Cy3-PEG by centrifugation, SARS-CoV-2 N protein (20 μL, 200 μg mL^−1^ in PBS) or streptavidin (20 μL, 200 μg mL^−1^ in 10 mM borate buffer) was added to Cy3-PEG-coated AuNPs solution (300 μL, 0.22 nM) and incubated for 20 min. High-purity separation of the dual-labeled nanoprobes was accomplished by repeated centrifugation. Detailed optimization of the nanoprobe construction can be found in Fig. S11 to S14 and ESI Methods.†

### Fabrication of serological LFIA test strips

The construction of the LFIA strip and the schematic illustration of the LFIA-based serological test procedure is present in Fig. 1. The conjugate release pad was soaked with borate buffer (10 mM, pH 9) containing sucrose (10%), BSA (1%) and Tween-20 (0.05%). Next, the equivolumetric mixture of N-protein-conjugated nanoprobe (optical density (OD) 2) and streptavidin-conjugated nanoprobe (OD 1) were dried on the conjugate release pad (10 mm × 4 mm) for 1 h. BIODOT dispenser was used to immobilize goat anti-human IgG (2 mg mL^−1^) and BSA-biotin (1.2 mg mL^−1^) on the test line (T line, 0.7 μL cm^−1^) and the control line (C line, 0.5 μL cm^−1^), respectively. Subsequently, each component of the serological LFIA sensor was dried at 37 °C, cut to a width of 4 mm and attached with a 2 mm overlap.

### Procedure for serological LFIA strip-based SARS-CoV-2 IgG detection

The samples for analytical validation were prepared by spiking the antibodies in normal human serum. The sample (10 μL) was added to a running buffer (90 μL, 0.5% tween-20 in PBS),^47^ and the mixture was loaded onto the port of the serological LFIA kits. From the strip constructed with our optimized conditions, the red-to-purple-colored lines with green fluorescence appeared on the strips approximately 8 minutes after sample loading without a sign of nanoprobe aggregation (Fig. S15†). The colorimetric and fluorescence signals from the strips were measured immediately afterwards.

### Acquisition and treatment of clinical samples of COVID-19 patients

All 73 human intravenous blood samples (43 positives and 30 negatives) were obtained from the Ewha Womans University Mokdong Hospital under the approval of the Institutional Review Board (IRB No. EUMC 2021-01-006). All patients were diagnosed as positive in RT-PCR. The clinical information of the patients is provided in Table S2.† Acquired blood samples were centrifuged to separate the serum (3500 rpm, 10 min) and stored in the deep freezer (−70 °C).

## Author contributions

S. K. Kim: investigation, methodology, data curation, formal analysis, writing – original draft. J. U. Lee: conceptualization, investigation, methodology, writing – reviewing & editing. M. J. Jeon: investigation, writing – reviewing & editing, S.-K. Kim: resources. S.-H. Hwang: resources. M. E. Hong: resources. S. J. Sim: supervision, funding acquisition, resources, writing – reviewing & editing, project administration.

## Conflicts of interest

There are no conflicts to declare.

## Supplementary Material

RA-013-D3RA04373H-s001
