# Supplementary material for: Bio-conjugated nanoarchitectonics with dual-labeled nanoparticles for a colorimetric and fluorescent dual-mode serological lateral flow immunoassay sensor in detection of SARS-CoV-2 in clinical samples
Source: RSC Adv. 2023 Sep 11;13(39):27225–32. doi: 10.1039/d3ra04373h (PMC10494995; doi:10.1039/d3ra04373h)
Supplement: RA-013-D3RA04373H-s001 [file RA-013-D3RA04373H-s001.pdf]

Electronic Supplementary Material (ESI) for RSC Advances.
This journal is © The Royal Society of Chemistry 2023

Supplementary Information

for

Bio-conjugated Nanoarchitectonics with Dual-labeled Nanoparticle for Colorimetric and Fluorescent Dual-mode Serological Lateral Flow Immunoassay Sensor in Detection of SARS-CoV-2 in Clinical Samples

Sang Ki Kim,[‡a] Jong Uk Lee,[‡b] Myeong Jin Jeon,[a] Soo-Kyung Kim,[c] Sang-Hyun Hwang,[d] Min Eui Hong[e] and Sang Jun Sim[*a]

[a.] Department of Chemical and Biological Engineering, Korea University, 145, Anam-ro, Seongbuk-gu, Seoul, 02841, Republic of Korea.

[b.] Department of Chemical Engineering, Sunchon National University, 225 Jungang-ro, Suncheon, Jeollanam-do, 57922, Republic of Korea

[c.] Department of Laboratory Medicine, Ewha Womans University Mokdong Hospital, Seoul, 07985, Republic of Korea

[d.] Department of Laboratory Medicine, Asan Medical Center, University of Ulsan College of Medicine, Seoul, 05505, Republic of Korea

[e.] Business Development, Kyung Nam Pharm.Co.,Ltd, 702 Eonju-ro, Gangnam-gu, Seoul, 06061, Republic of Korea

‡These authors contributed equally to this work.

*Authors to whom correspondence should be addressed: simsj@korea.ac.kr

Supplementary Methods

Optimization of the dual-labeled nanoprobes

The concentration of Cy3-labeled thiolated PEG, pH of the colloidal gold solution, and concentration of SARS-CoV-2 antigens are essential for stabilizing the dual-labeled nanoprobe and sensitivity of MEF based dual-mode LFIA sensor. The concentration of Cy3-labeled thiolated PEG was optimized as follows: Different volumes of Cy3-PEG-SH (200 µg/mL) from 5 µL to 100 µL were added to 1mL of gold solution (0.22 nM) and incubated overnight. After centrifugation, the probe showing gray color (from 35 µL to 100 µL) was removed. Then 10 µL of each Cy3-labeled AuNPs (0.025 nM) were dried on the conjugate pad with running buffer loaded. After taking out an assay that did not flow, the remaining nanoprobes were placed in the wells of a 96-well microplate. Subsequently, fluorescence intensity was measured by a microplate reader (Tecan, GENios).

The optimal pH of the colloidal gold solution and concentration of the SARS-CoV-2 antigen was determined using the T/C ratio of the colorimetric assay. To optimize the pH of the gold solution, the pH varied from 5 to 11. An identical amount of SARS-CoV-2 antigen was then added to each gold solution. The mixture was incubated for 20 min at room temperature with vigorous stirring. After centrifugation, each nanoprobe was dried on a conjugate pad with the loaded sample buffer. The T/C ratio of the colorimetric signal was evaluated using ImageJ. Optimization of the concentration of SARS-CoV-2 antigen was conducted in the same way as pH optimization, with the volume ranging from 1 µL to 24 µL.

Selection of chemical buffer for pre-treatment of the strips and running buffer

Pretreatment of the conjugate pads and optimization of the running buffer is essential for the optimal release and stability of the nanoprobes. Pretreatment of the conjugate pad was prepared as follows: (1) borate buffer (10 mM, pH 7.4) containing 0.05% (v/v) Tween-20 with 0%, 1%, 2% (w/v) BSA; (2) borate buffer (10 mM, pH 7.4) containing 0.05% (v/v) Tween-20 with 0%, 1%, 2% (w/v) BSA, 5%, 10%, 20% (w/v) sucrose; (3) PBS (10 mM, pH 7.4) containing 0.05% (v/v) Tween-20 with 0%, 1%, 2% (w/v) BSA; and (4) PBS (10 mM, pH 7.4) containing 0.05% (v/v) Tween-20 with 0%, 1%, 2% (w/v) BSA and 5%, 10%, 20% (w/v) sucrose. Conjugate pads were soaked in the buffer for 1 h. Additionally, the dual-labeled nanoprobes were loaded onto the conjugate pad and dried for 1 h. Upon optimizing the adequate buffer for pretreatment, the nanoprobes on the conjugate pad soaked in PBS buffer turned gray. Simultaneously, fluorescence signals could not be observed due to aggregation. When a buffer containing 2% (w/v) BSA and 20% (w/v) sucrose was used, the flow of the assay could not be observed because the nanoprobes were stocked on the conjugate pad. Finally, by observing the lateral flow speed and signal intensity, borate buffer (10 mM, pH 7.4) containing 0.05% (v/v) Tween-20, 1% (w/v) BSA, and 10% (w/v) sucrose was selected as the optimal buffer for pre-treating the conjugate pad. In addition, the running buffer was optimized with different concentration of Tween-20 (v/v) in PBS to achieve a smooth flow. The running buffer containing 0.5% (v/v) Tween-20 exhibited excellent performance, demonstrating the highest

color contrast signals on the test lines.

Comparison of various types of SARS-CoV-2 antigen-antibody interactions

Five different types of SARS-CoV-2 antigens (N, S, S1, S2, S-RBD) were incubated with Cy3 labeled thiolated PEG conjugated AuNPs to compare the affinity of each protein. After fabricating 5 different dual-labeled nanoprobes, the specificity and the sensitivity were evaluated by measuring RPI using ImageJ. (Fig. S2 & S3). Since nucleocapsid protein is more stable than spike protein from mutations, nucleocapsid antigen-conjugated dual-labeled nanoprobes only bound to nucleocapsid antibodies demonstrating excellent specificity. In contrast, spike and spike subunits cross-react with each other showing little peak intensity. In addition, nucleocapsid protein conjugated dual-labeled nanoprobes-based LFIA sensor showed the highest sensitivity by detecting corresponding antibodies as low as 1 ng/mL, followed by spike RBD, Spike, S2, and S1.

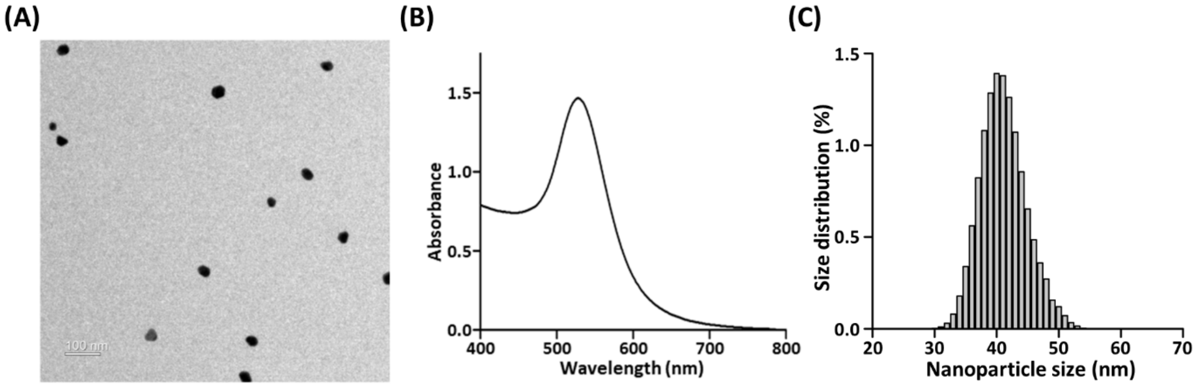

Fig. S1 Characterization of AuNPs. (A) TEM image, (B) UV-vis spectra, and (C) NTA measurement of size distribution.

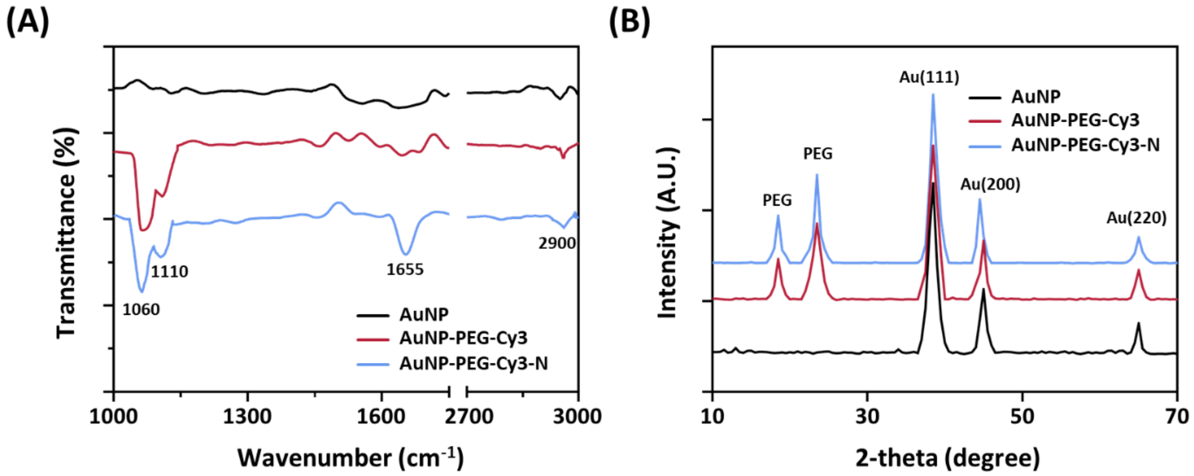

Fig. S2 Characterization of nanoprobes. (A) FT-IR spectrum and (B) XRD spectrum of AuNPs modified with Cy3 and SARS-CoV-2 N antigen.

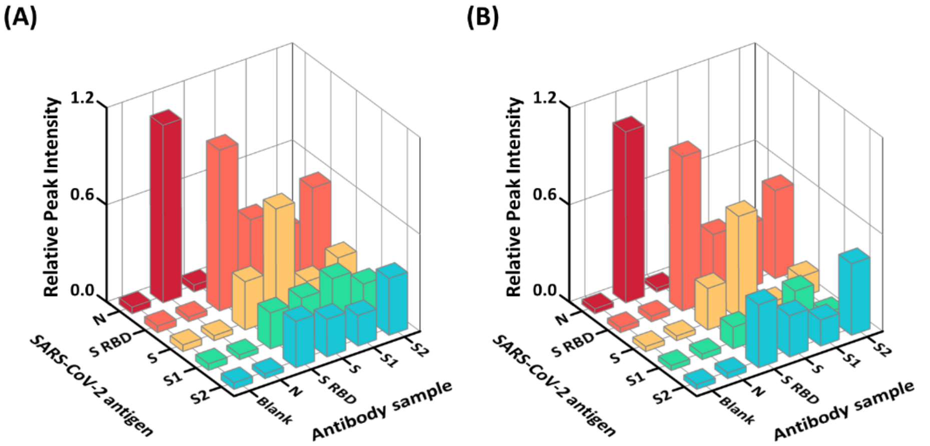

Fig. S3 Comparison of 5 different SARS-CoV-2 antigen-antibody affinities by measuring specificity. (A) Colorimetric signal intensity and (B) Fluorescence signal intensity.

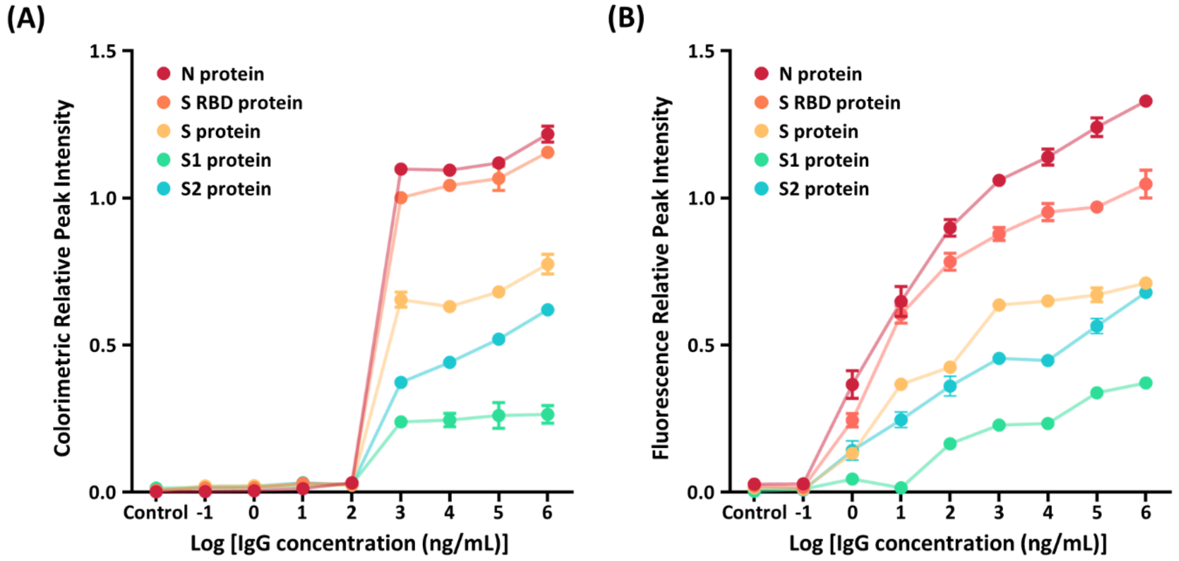

Fig. S4 Comparison of 5 different SARS-CoV-2 antigen-antibody affinities by measuring sensitivity. (A) Colorimetric signal intensity and (B) Fluorescence signal intensity.

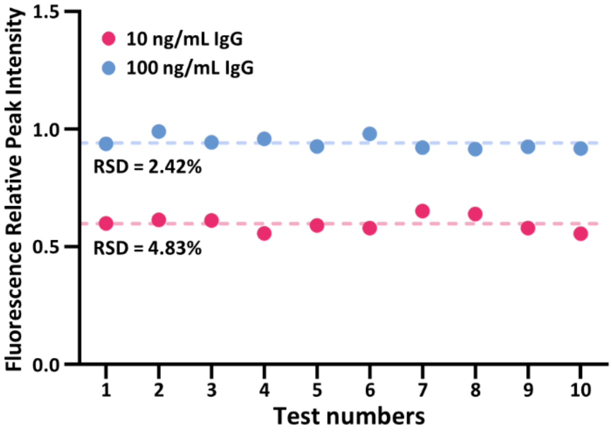

Fig. S5 Reproducibility of fluorescence signals for 100 ng/mL and 10 ng/mL IgG concentrations.

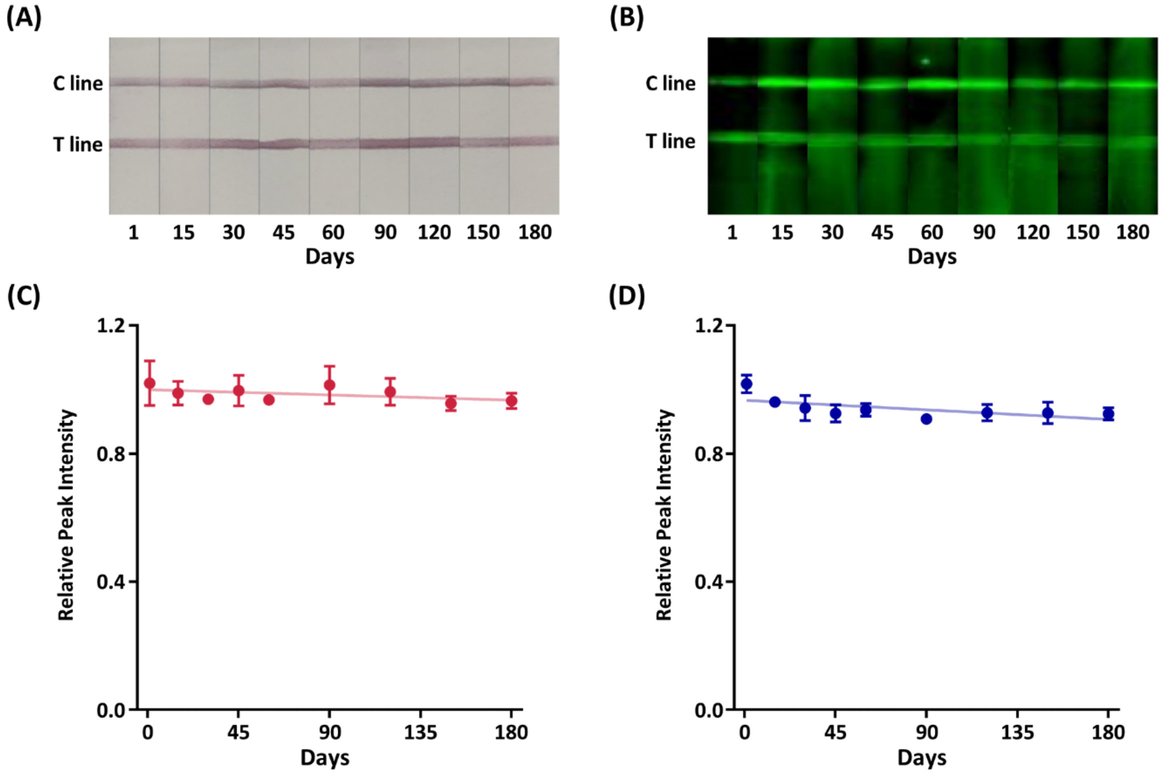

Fig. S6 Stability tests of the MEF-based dual-mode serological LFIA. Each strip was stored at room temperature for a period of 1-180 days. (A and B) Colorimetric images fluorescent images of each test, (C and D) Relative peak intensity of color and fluorescence signals of each test. Error bars indicate the standard deviation from three independent experiments.

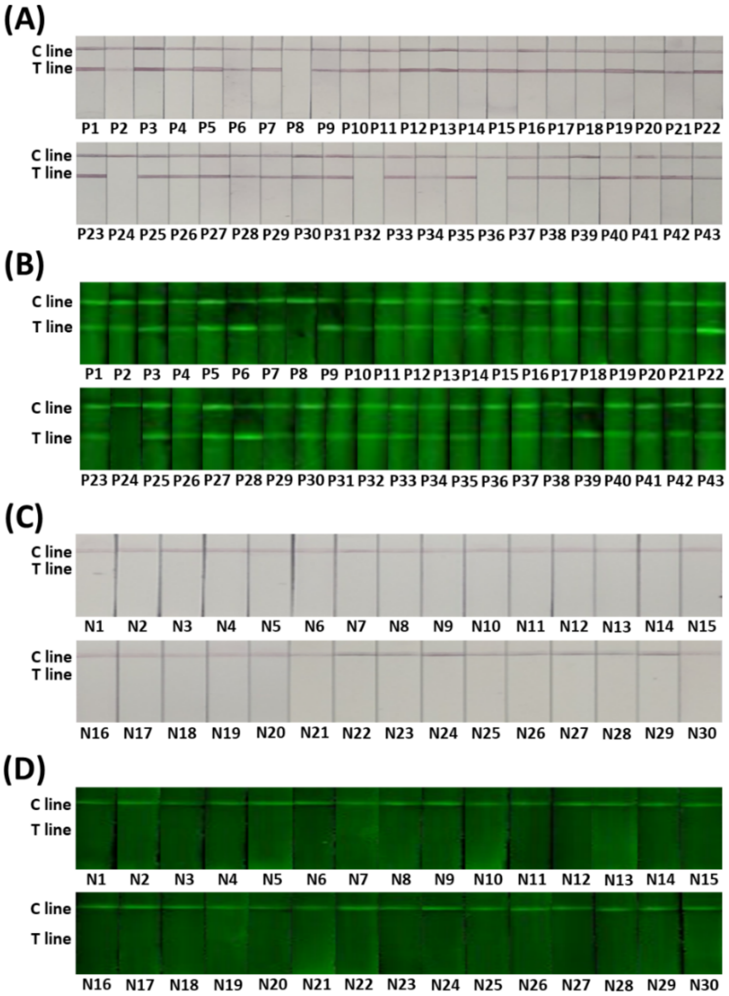

Fig. S7 MEF-based dual-mode serological LFIA with 73 clinical samples. (A) Colorimetric and (B) fluorescent images of 43 serum samples of patients. (C) Colorimetric and (D) fluorescent images of 30 negative serum samples.

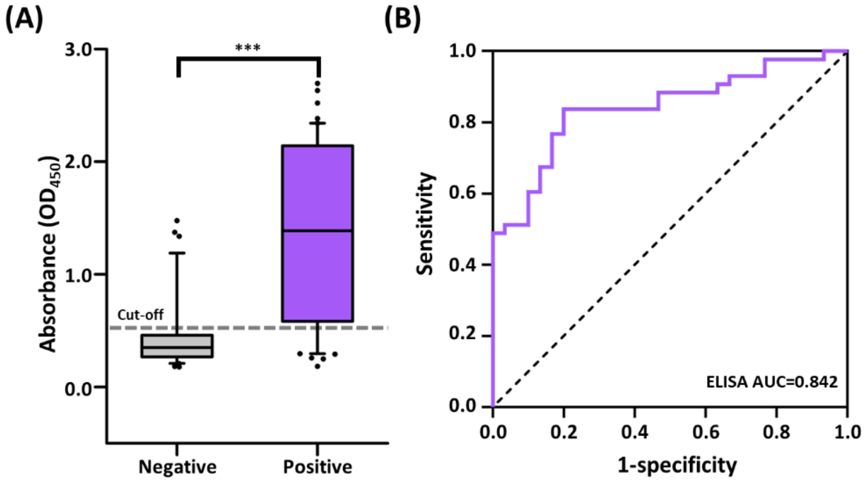

Fig. S8 ELISA with 73 clinical serum samples: 43 COVID-19 positive serum and 30 negative serum samples (A) scatter plot of 43 COVID-19 positive serum and 30 negative serum samples. ***p<0.0001. (B) ROC curve analysis of 73 serum samples to assess the detection capacity of the ELISA.

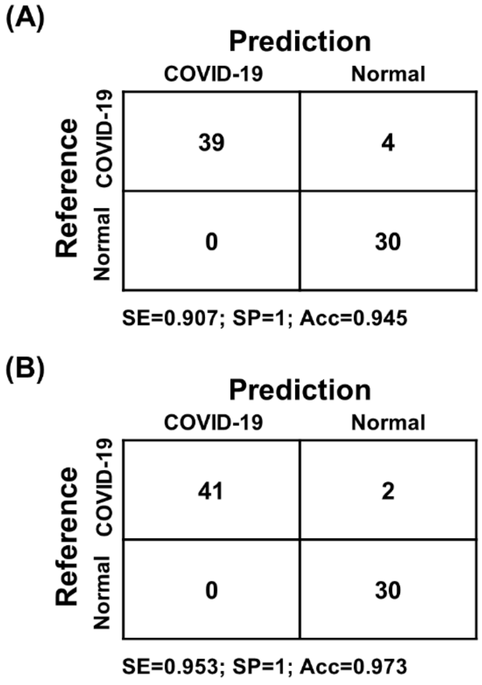

Fig. S9 Confusion matrix of (A) colorimetric and (B) fluorescent assay of MEF-based dual-mode serological LFIA sensor. SE, sensitivity; SP, specificity; Acc, accuracy.

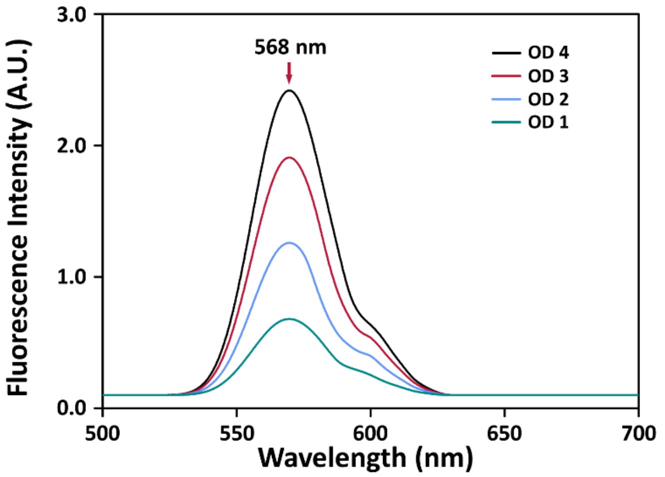

Fig. S10 Representative fluorescence spectrum of nanoprobe with different OD.

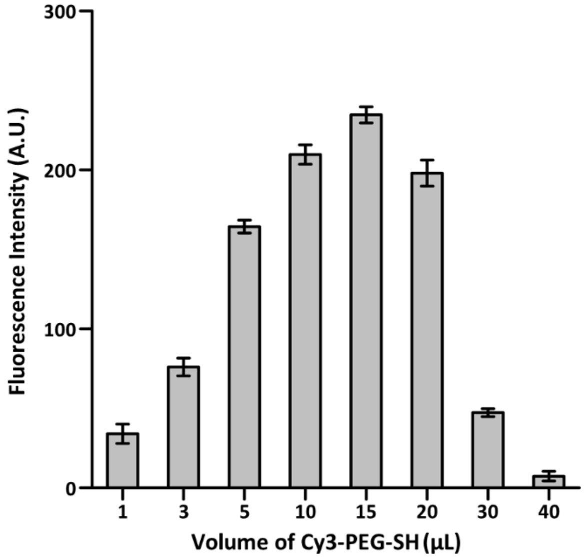

Fig. S11 Optimizing the concentration of Cy3-PEG-SH on 40 nm AuNPs.

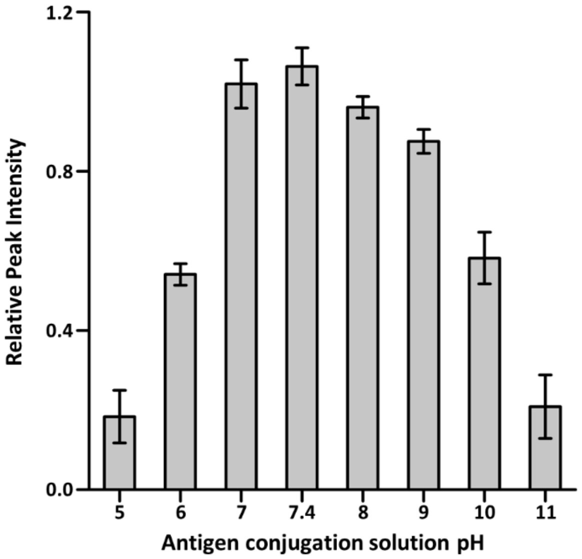

Fig. S12 Optimizing pH of solution for SARS-CoV-2 antigen conjugation.

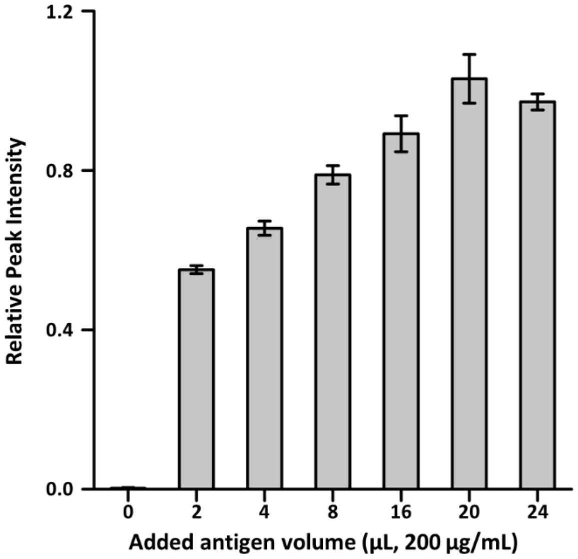

Fig. S13 Optimizing the concentration of SARS-CoV-2 antigen for fabrication of nanoprobes.

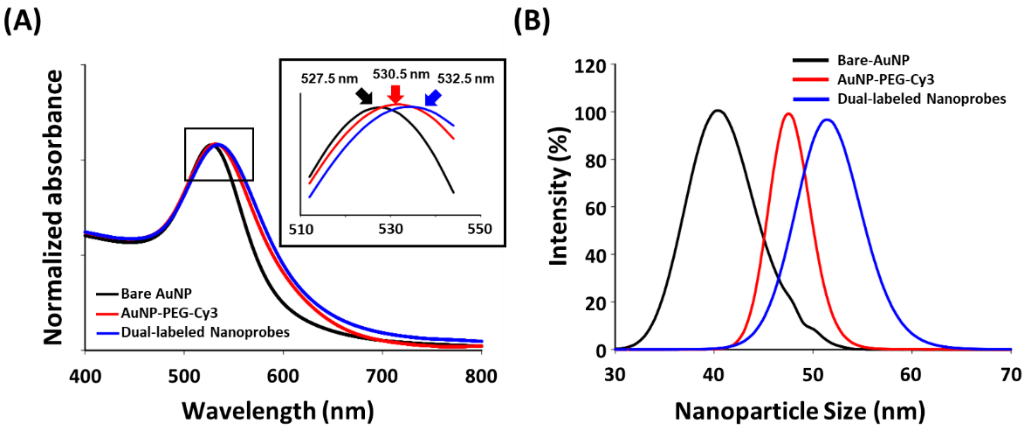

Fig. S14 Characterization of nanoprobes. (A) UV-Vis spectra of bare AuNPs, Cy3-PEG conjugated AuNPs and dual-labeled nanoprobes. (B) Size distribution of bare AuNPs, Cy3-PEG conjugated AuNPs, and dual-labeled nanoprobes measured by NTA.

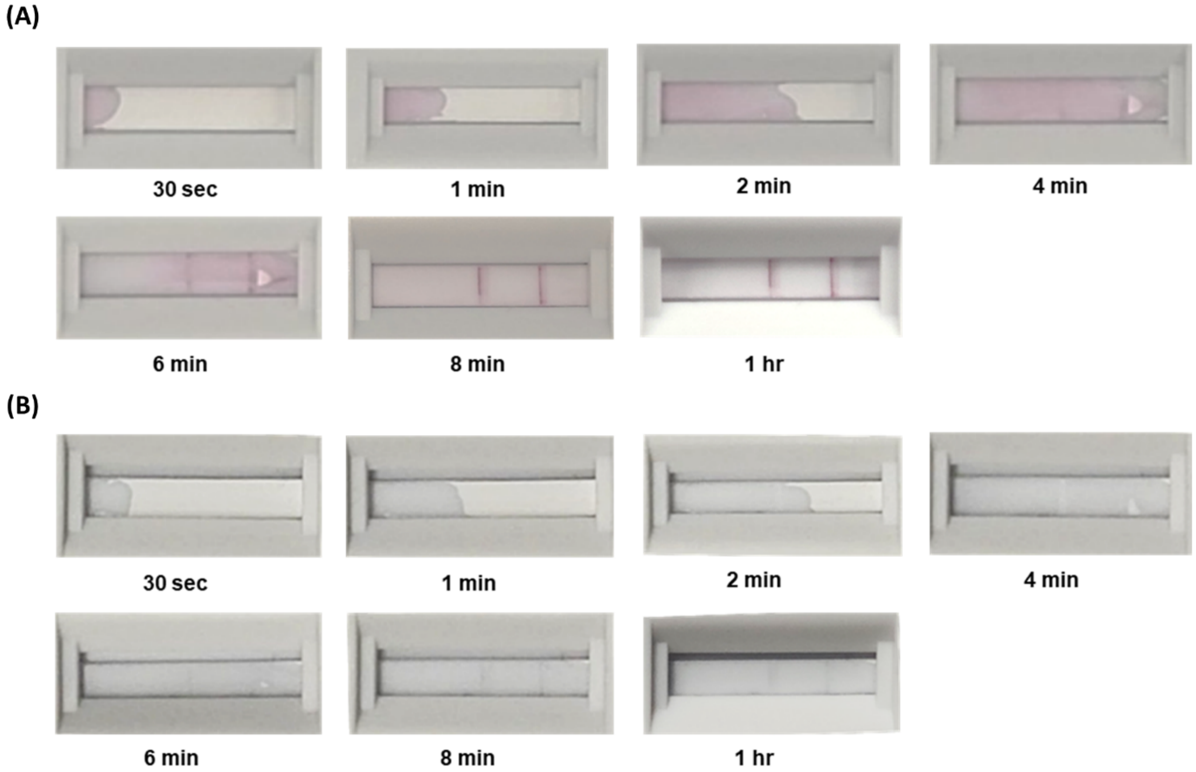

Fig. S15 Consecutive images of colorimetric LFIA after loading a positive sample (1 μg/mL anti-N IgG). (A) Appearance of colorimetric signal within 8 minutes when nanoprobes are stable. (B) Aggregated nanoprobes cannot flow along with the sample, showing no visible lines.

Table S1. A comparison of the MEF-based dual-mode LFIA sensor with the recently reported assay of serological SARS-CoV-2 detection.

Samples	Analytes	Method	Detection time [min]	Limit of detection	Sensitivity [%]	Specificity [%]	Ref.
Serum	IgM and IgG	Fluorescence	10	0.236 μg/ml (IgM)<br>0.125 μg/ml (IgG)	78 (IgM)<br>95 (IgG)	/	(1)
Serum	IgG	Colorimetric	15-20	0.8 mg/ml	69.1	100	(2)
Human blood	IgM and IgG	Fluorescence	15	48.84 ng/ml	88.7	90.6	(3)
Serum and blood	IgM and IgG	Colorimetric	10	20 ng/ml (IgM)<br>5 ng/ml (IgG)	93.33	97.34	(4)
Serum	IgM and IgG	Colorimetric	10	60 ng/ml (IgM)<br>20 ng/ml (IgG)	94.74	96.23	(5)
Serum	IgM and IgG	Colorimetric	/	/	88.56	97.42	(6)
Serum and plasma	IgM and IgG	Colorimetric	10-15	0.8 μg/ml (IgM)<br>0.6 μg/ml (IgG)	90	96.6	(7)
Serum	IgG	Fluorescence	8	48.84 ng/ml	/	/	(8)
Serum	IgM and IgG	Fluorescence	15	/	97.37	95.54	(9)
Serum	IgG	Colorimetric/ Fluorescence	8	1 ng/ml	95.3	100	This work

Table S2. Clinical sample information of the COVID-19 patients.

Patient No.	Gender	Age	Sample acquisition date (yymmdd)	Dual-mode LFIA (Color)	Dual-mode LFIA (Fluorescent)
P1	Female	77	210512	+	+
P2	Female	70	210427	+	+
P3	Male	50	210529	+	+
P4	Male	82	210519	+	+
P5	Male	61	210517	+	+
P6	Female	26	210606	+	+
P7	Male	44	210807	+	+
P8	Male	35	210815	-	-
P9	Male	66	210804	+	+
P10	Male	70	210807	+	+
P11	Female	75	210823	+	+
P12	Female	68	210824	+	+
P13	Female	93	210824	+	+
P14	Male	36	210808	+	+
P15	Male	47	210829	+	+
P16	Female	66	220228	+	+
P17	Female	75	220228	+	+
P18	Male	91	220228	+	+
P19	Female	56	220228	+	+
P20	Male	48	220228	+	+
P21	Male	73	220228	+	+
P22	Female	71	220428	+	+
P23	Male	65	220428	+	+
P24	Male	78	220428	-	-
P25	Female	92	220428	+	+
P26	Male	78	220504	+	+
P27	Female	63	220428	+	+

P28	Male	80	220428	+	+
P29	Male	53	220429	+	+
P30	Male	82	220425	+	+
P31	Male	63	220501	+	+
P32	Female	62	220428	-	+
P33	Male	94	220428	+	+
P34	Male	76	220428	+	+
P35	Male	87	220504	+	+
P36	Male	89	220503	-	+
P37	Male	45	220428	+	+
P38	Female	68	220504	+	+
P39	Female	51	220428	+	+
P40	Female	27	220427	+	+
P41	Male	88	220429	+	+
P42	Female	61	220429	+	+
P43	Male	65	220427	+	+

Supplementary Reference

[1] R. Chen, C. Ren, M. Liu, X. Ge, M. Qu, X. Zhou, M. Liang, Y. Liu and F. Li, *ACS Nano*, 2021, **15**, 8996.

[2] T. Wen, C. Huang, F.-J. Shi, X.-Y. Zeng, T. Lu, S.-N. Ding and Y.-J. Jiao, *Analyst*, 2020, **145**, 5345.

[3] Z. Li, Y. Yi, X. Luo, N. Xiong, Y. Liu, S. Li, R. Sun, Y. Wang, B. Hu and W. Chen, *J. Med. Virol.*, 2020, **92**, 1518.

[4] Z. Wang, Z. Zheng, H. Hu, Q. Zhou, W. Liu, X. Li, Z. Liu, Y. Wang and Y. Ma, *Lab Chip*, 2020, **20**, 4255.

[5] C. Chen, H. Hu, X. Li, Z. Zheng, Z. Wang, X. Wang, P. Zheng, F. Cui, G. Li and Y. Wang, *IEEE Trans. Nanobiosci.*, 2021, **21**, 37.

[6] K. Li, C. Tong, X. Ha, C. Zeng, X. Chen, F. Xu, J. Wang, H. Du, Y. Chen and J. Cai, *BMC Infect. Dis.*, 2021, **21**, 860.

[7] A. Ahmadi, Z. Mirzaeizadeh and K. Omidfar, Monoclon. *Antibodies Immunodiagn. Immunother.*, 2021, **40**, 210.

[8] Z. Li, A. Wang, J. Zhou, Y. Chen, H. Liu, Y. Liu, Y. Zhang, P. Ding, X. Zhu and C. Liang, *Int. J. Mol. Sci.*, 2022, **23**, 6225.

[9] C. Wang, D. Shi, N. Wan, X. Yang, H. Liu, H. Gao, M. Zhang, Z. Bai, D. Li, E. Dai, Z. Rong, S. Wang, *Analyst*, 2021, **146**, 3908.
